# Cotton roots are the major source of gossypol biosynthesis and accumulation

**DOI:** 10.1186/s12870-020-2294-9

**Published:** 2020-02-27

**Authors:** Tianlun Zhao, Qianwen Xie, Cong Li, Cheng Li, Lei Mei, John Z. Yu, Jinhong Chen, Shuijin Zhu

**Affiliations:** 1 Department of Agronomy, Zhejiang University, Hangzhou, 310058 Zhejiang China; 2 USDA-ARS, Southern Plains Agricultural Research Center, College Station, TX 77845 USA

**Keywords:** Cotton, Gossypol biosynthesis, Plant grafting, Organ culture, Gene expression profiling

## Abstract

**Background:**

Gossypol is a specific secondary metabolite in *Gossypium* species. It not only plays a critical role in development and self-protection of cotton plants, but also can be used as important anti-cancer and male contraceptive compound. However, due to the toxicity of gossypol for human beings and monogastric animals, the consumption of cottonseeds was limited. To date, little is known about the gossypol metabolism in cotton plants.

**Results:**

In this study, we found that cotyledon was the primary source of gossypol at the seed germination stage. But thereafter, it was mainly originated from developing roots. Grafting between glanded and glandless cotton as well as sunflower rootstocks and cotton scion revealed that gossypol was mainly synthesized in the root systems of cotton plants. And both glanded and glandless cotton roots had the ability of gossypol biosynthesis. But the pigment glands, the main storage of gossypol, had indirect effects on gossypol biosynthesis. In vitro culture of root and rootless seedling confirmed the strong gossypol biosynthesis ability in root system and the relatively weak gossypol biosynthesis ability in other organs of the seedling. Expression profiling of the key genes involved in the gossypol biosynthetic pathway also supported the root as the major organ of gossypol biosynthesis.

**Conclusions:**

Our study provide evidence that the cotton root system is the major source of gossypol in both glanded and glandless cottons, while other organs have a relatively weak ability to synthesize gossypol. Gossypol biosynthesis is not directed related to the expression of pigment glands, but the presence of pigment glands is essential for gossypol accumulation. These findings can not only clarify the complex regulation network of gossypol metabolism, but it could also accelerate the crop breeding process with enhanced commercial values.

## Background

Cotton (*Gossypium* spp.) is one of the most important economic crops in the world. Cotton not only produces natural fiber for textile industry, but it also provides a large quantity of cottonseeds which contain high-quality protein and oil [1]. It is estimated that every kilogram fiber yield is coupled with 1.65 kg cottonseeds, which contain approximately 21% oil and 23% protein [2]. However, cottonseeds cannot be used directly due to the presence of gossypol, a toxic substance to human beings and monogastric animals [3]. On the other hand, gossypol plays an important role in self-protection of cotton plants [4–6].

Gossypol was first characterized by Adams et al. in 1938 through a series of classic studies [7]. It is a polyphenolic aldehyde which constitutes 20–40% of the pigment glands weight and accounts for 0.4–1.7% of the whole cottonseed kernel. As a phytoalexin, gossypol provides constitutive and inducible resistance against pests and pathogens [8–13]. Besides, gossypol can be used as anti-cancer [14–16], anti-bacterial [17, 18] and male contraceptive reagent [19–21]. There are two different enantiomers of gossypol, (+)-gossypol and (−)-gossypol. Based on previous studies, the biological activity of (−)-gossypol is stronger than that of (+)-gossypol [22–24]. Additionally, the difference in ratio of enantiomers in cottonseeds might affect the poultry production when they were used as poultry feed [25].

Several key genes involved in the pathway of the gossypol biosynthesis have been identified and characterized, such as terpene synthase genes, *GhTPS1* and *GhTPS2* [26], and *hmg1* and *hmg2* that encode the limiting enzymes of isoprenoid biosynthesis pathway [27]. Other key genes, which usually exist as gene families, of the gossypol biosynthesis pathway, include *CAD1*-*A*, *CAD1*-*C2* [28] and *cdn1*-*C4* [29]. In addition, *GaCYP706B1* was isolated as the gene encoding cadinene-8-hydroxylase, the key enzyme in the hemigossypol biosynthesis [30]. *GaWRKY1*, a transcription factor involved in regulation of sesquiterpene biosynthesis which affects the expression of *CAD*-*1*, has also been identified [31]. A recent study characterized four key genes in the downstream of gossypol biosynthetic pathway [32]. With tremendous progress in molecular biology, gene transformation, virus-induced gene silencing (VIGS) and CRISPR/Cas9 system have been applied to study cotton traits [2, 33, 34]. These molecular tools together with other genetic and physiological approaches provide a good opportunity to dissect the complex mechanism underlying gossypol metabolism.

To make an apparent relationship between pigment gland and gossypol content in cotton plants is very important. Punit et al. found that different genotypes had an impact on the distribution of pigment glands [35]. And the gossypol content in cotton was closely related to the genotypes of pigment glands. Singh and Weaver proposed that the gossypol content was highly correlated with the number of pigment glands [36], except in *Gossypium somalense Huntch*, which had almost no gossypol in seed although the presence of normal pigment glands [37]. Silencing *CYP706B1*, a key gene in the gossypol biosynthesis pathway, Ma et al. found that the gossypol content significantly decreased but the pigment glands still formed as normal plant, and eliminating the pigment glands through VIGS led to decline greatly in gossypol content [33]. Therefore, the relationship between gossypol and pigment glands was related but remains unclear, which need more research to clarify.

Understanding the metabolism of gossypol is essential for developing cotton cultivars with low gossypol seeds that have a wider utilization potential. However, only few studies have been carried out to elucidate the biosynthesis and transportation of gossypol. Smith proposed that gossypol was synthesized in cotton root based on in vitro root culture [38], but no study has been reported on whether or not other tissues also have the ability to synthesize gossypol. To clearly define the tissues/organs involved in gossypol biosynthesis and transportation in cotton plants is not only important for understanding the mechanism of gossypol biosynthesis, but also helpful for breeding new cotton cultivars with low gossypol content in cottonseeds by genetic modification.

In the present study, the biosynthesis and transportation of gossypol in cotton plant, the relationship between gossypol and pigment glands in cotton plants, and the capacity of gossypol biosynthesis among the different organs and different isogenic lines of glanded and glandless cottons were investigated by using the methods of plant grafting, organ culture and gene expression profiling, which may find some useful information for scientists to manipulate the gossypol biosynthesis in cotton plants and develop a new cotton cultivar only without gossypol in the cottonseeds.

## Results

### Gossypol change in organs of glanded and glandless cotton seedlings

**Fig. 1:**
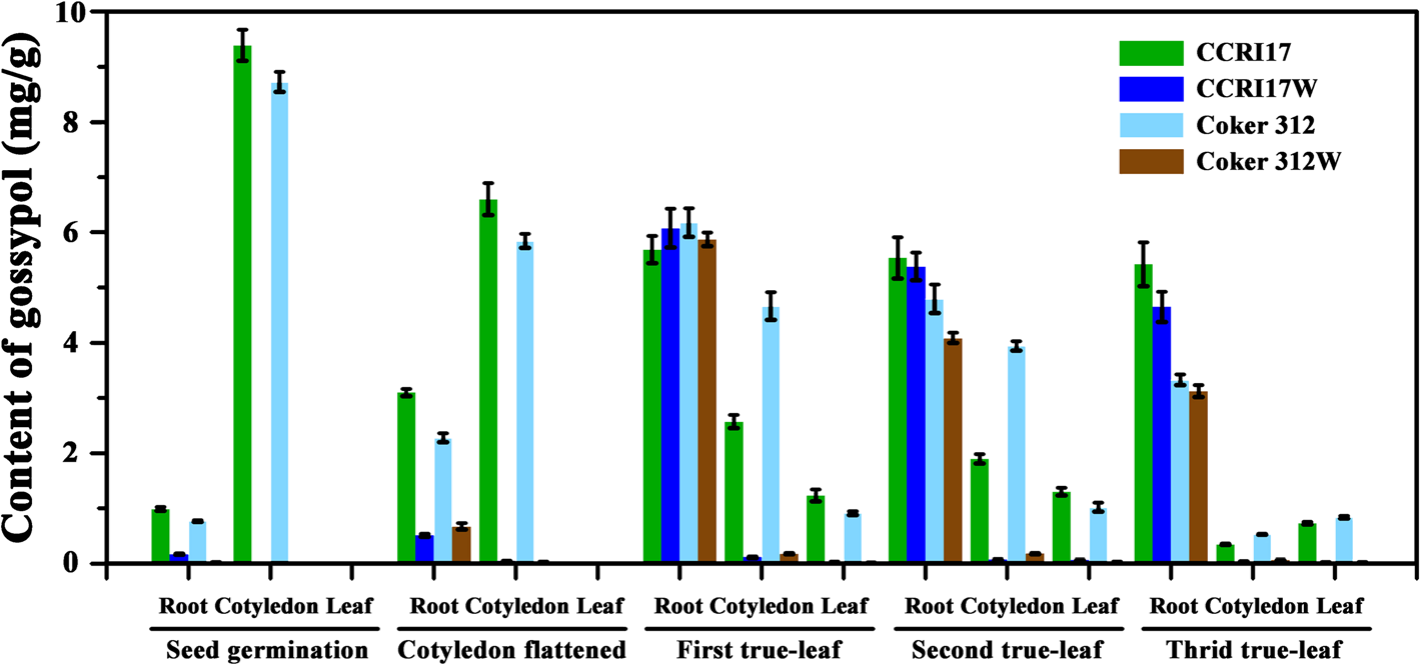
Dynamic changes of gossypol content in tissues of the CCRI17, CCRI17W, Coker 312 and Coker 312 W at the stages of seed germination, cotyledon flattened, seedlings with first, second and third true-leaf. No leaf samples were taken at seed germination and cotyledon flattened stages. Each sample was carried out in three biological repetitions

Two pairs of near isogenic lines (NILs), CCRI17 (glanded) vs CCRI17W (glandless) and Coker 312 (glanded) vs Coker 312 W (glandless), were planted in greenhouse. The gossypol contents in root, cotyledon and leaf of their seedlings at different stages of seedling were analyzed (Fig. 1).

In cotyledon, the gossypol content in glanded cotton was always higher than that in their glandless NILs at all stages. Specifically, at the seed germination stage, the gossypol contents in the glanded cotton, CCRI17 (9.384 mg/g) and Coker 312 (8.722 mg/g), were much higher as compared with their corresponding glandless NILs, CCRI17W (0.011 mg/g) and Coker 312 W (0.008 mg/g). At the subsequent stages, the gossypol contents in CCRI17 and Coker 312 were gradually decreased. At the third true-leaf stage, their gossypol contents were reduced to 0.345 and 0.528 mg/g, respectively. However, the gossypol contents increased with a peak at the first true-leaf stage in CCRI17W (0.116 mg/g) and second true-leaf stage in Coker 312 W (0.177 mg/g).

Similarly, the gossypol content in the leaves of glanded cotton was much higher than their glandless NILs. It was increased with the plant development and reached the peak at the second true-leaf stage in both pairs of NILs. However, the gossypol contents in the leaves of glandless NILs were consistently low. The peak contents were 0.007 and 0.004 mg/g for CCRI17W and Coker 312 W, respectively. While the peak contents were 0.130 and 0.102 mg/g for CCRI17 and Coker 312, respectively, which were 18 times higher than their glandless NILs.

At the seed germination stage, the gossypol contents in the roots of CCRI17 and Coker 312 were 0.986 and 0.768 mg/g, respectively, which were much higher as compared with their glandless NILs, CCRI17W (0.153 mg/g) and Coker 312 W (0.023 mg/g). With the growth of seedlings, the gossypol contents in roots were increased and reached the peak levels at the first true-leaf stage in all four genotypes. At the peak stage, the differences of gossypol contents between the glanded and glandless NILs were not significant, implying that the biosynthesis and accumulation of gossypol in glandless cotton roots were as strong as their glandled NILs. The general trends of root gossypol contents, for both the glanded and glandless genotypes, were significantly increased from seed germination to the first true-leaf stage and then remain unchanged or slightly decreased from the first true-leaf to the third true-leaf stage (Fig. 1).

### Plant grafting

To investigate the effect of root system on gossypol biosynthesis, grafting with different combinations of scion (glanded cotton or glandless cotton) and rootstock (glanded, glandless, or sunflower) were performed (Additional file 1: Figure S1).

All the graft combinations with cotton scion and cotton rootstock produced normal plants from which cottonseeds were harvested. However, the grafted plants with the combination of cotton scions and sunflower rootstocks grew very weak and died after 2 weeks, except those with the regenerated roots coming from the cotton scion.

#### Grafting between glanded and glandless cottons

The size and density of pigment glands in leaves and seeds were measured and compared between the grafted and normal plants (Additional file 2: Table S1). No significant difference in the size and density of pigment glands was observed among the normal glanded plants, the grafted glanded scions with glanded or glandless rootstocks. Similar to normal glandless plants, no pigment gland was observed on the grafted glandless scions with glandless or glanded rootstocks.

**Fig. 2:**
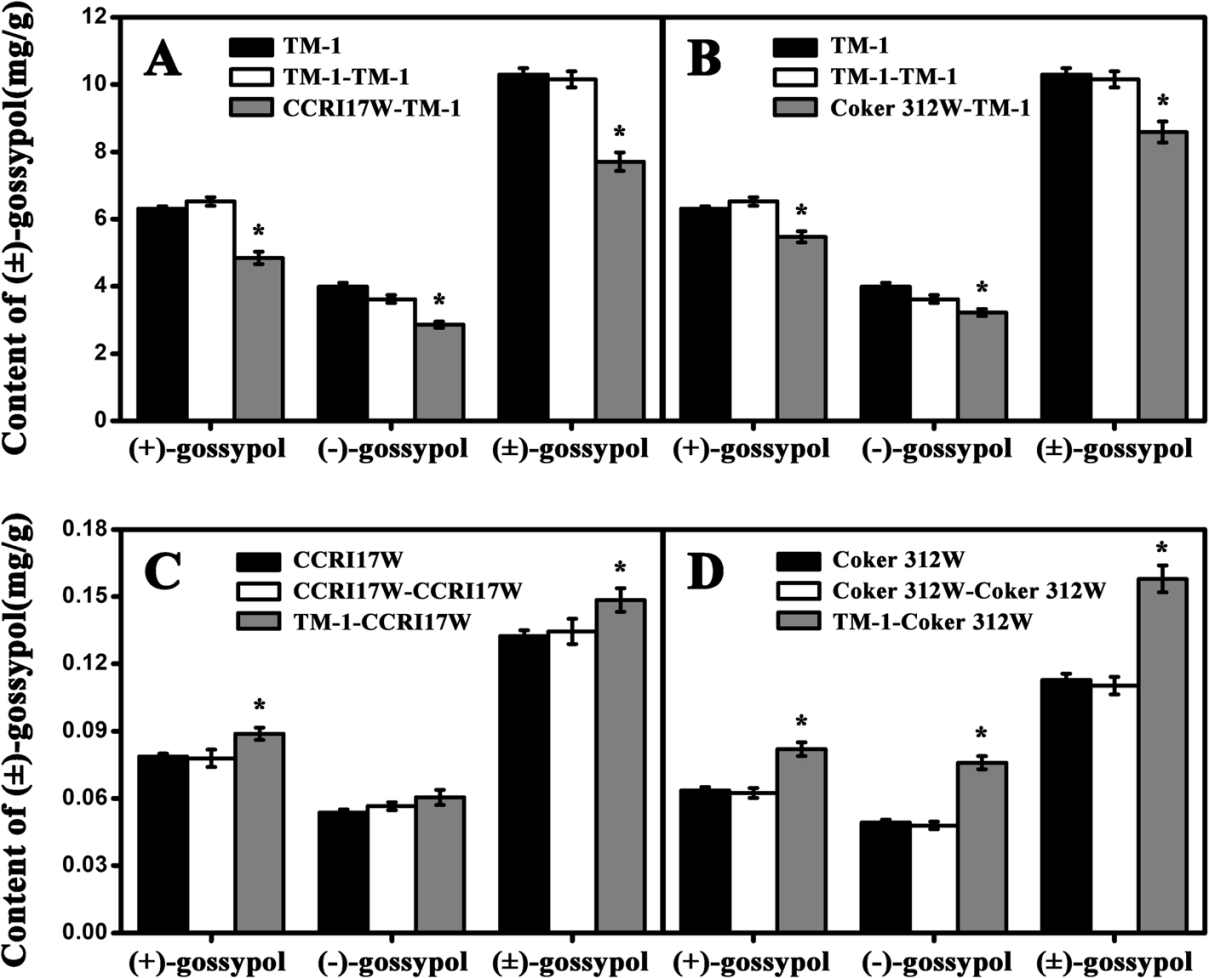
The (±)-gossypol contents in cottonseeds in normal and grafted plants. The black bars showed the gossypol contents in normal plants cottonseeds, the white bars showed the gossypol contents in grafted plant cottonseeds with scion and rootstock coming from same cultivar and the gray bars showed the gossypol contents in grafted plant cottonseeds with scion and rootstock coming from different cultivars. The former name of the graft combination was the rootstock cultivar name and the latter name was the scion cultivar name. Each sample was carried out in three biological repetitions. * indicates significant difference, *P* < 0.05

Gossypol contents in cottonseeds were greatly varied among the normal plants, self-grafted plants and grafted plants with rootstocks of different types of pigment gland. The gossypol contents in cottonseeds from self-grafted plants showed no difference as compared with normal plants in all four genotypes (Fig. 2), which indicated that grafting had no effect on the gossypol content. However, for the grafting combination using dominant glandless cotton (CCRI17W) as rootstock, the gossypol content in the cottonseeds of glanded cotton TM-1 scions was significantly lower than those in the self-grafted and normal TM-1 cottonseeds. Compared with normal TM-1 cottonseeds, the content of (+)-gossypol, (−)-gossypol and (±)-gossypol were decreased by 25.76, 20.96 and 24.05%, respectively (Fig. 2a). Similar phenomenon was observed in the cottonseeds harvested from the grafting combination of recessive glandless cotton (Coker 312 W) rootstock and TM-1 scion, the contents of (+)-gossypol, (−)-gossypol and (±)-gossypol decreased by 16.18, 10.97 and 15.34%, respectively (Fig. 2b) as compared with normal cottonseeds. On the contrary, when the glanded cotton, TM-1, was used as rootstocks, cottonseeds from the grafted glandless plants had a significant higher gossypol contents than cottonseeds from their self-grafted and normal plants. Compared with normal cottonseeds, the contents of (+)-gossypol, (−)-gossypol and (±)-gossypol increased by 14.09, 7.95 and 10.45% in the cottonseeds of CCRI17W (Fig. 2c), while it was 31.39, 58.46 and 43.16%, respectively in the cottonseeds of Coker 312 W (Fig. 2d).

#### Grafting between sunflower and glanded cotton

To further investigate the ability of the cotton root system to synthesize gossypol, we grafted glanded cotton scions (TM-1) on the sunflower rootstocks which are unable to synthesize gossypol. Due to the isolation of species, the cotton scions survived on the sunflower rootstock for only about 2 weeks, except those scions with regenerated roots (Additional file 3: Figure S2).

**Fig. 3:**
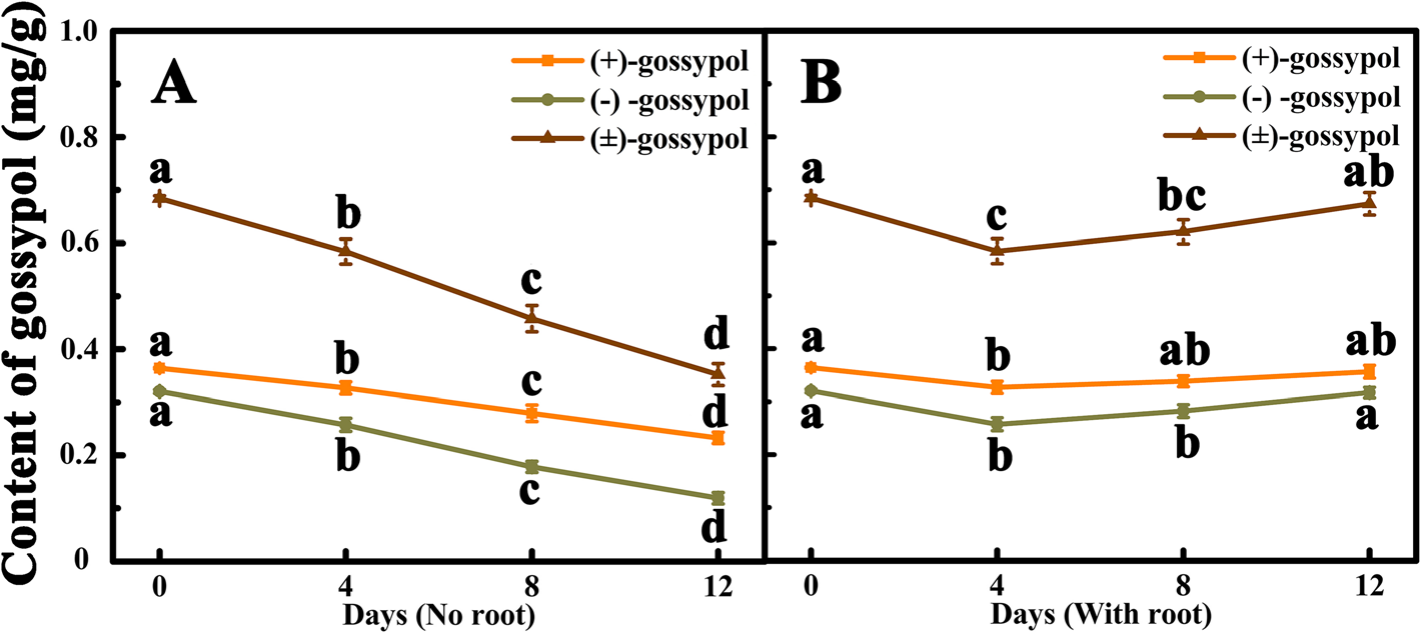
Dynamic changes of (±)-gossypol content in the leaves of TM-1 scions after grafting on the rootstock of sunflower. **a** Gossypol content change in the leaves of TM-1 scions without regenerated root. **b** Gossypol content in the leaves of TM-1 scions with regenerated root. Each sample was carried out in three biological repetitions. Different letters indicate significant difference at the different times, *P* < 0.05

The dynamic change of (±)-gossypol in scion leaves were analyzed. At the day of grafting, the contents of (+)-gossypol, (−)-gossypol and (±)-gossypol were 0.364, 0.320, and 0.684 mg/g, respectively. Four days after grafting, the contents of (±)-gossypol significantly decreased in both scions with and without regenerated roots (Fig. 3), and then continued the significant decrease in the scions without regenerated roots. At 12 days after grafting, the contents of (+)-gossypol, (−)-gossypol and (±)-gossypol in the scions without regenerated roots were significantly reduced by 36.00, 62.81 and 48.56%, respectively, compared with that of day 0 (Fig. 3a). In contrast, from day 4 to day 12 after grafting, the contents of (+)-gossypol, (−)-gossypol, and (±)-gossypol in the leaves of scions with regenerated roots were increased and reached the levels of day 0 (Fig. 3b).

### Gossypol change in cultured organs in vitro

To further investigate the role of roots in gossypol biosynthesis, we separated the geminated seedlings of the two pairs of NILs into two parts: root and rootless seedlings (including cotyledon and hypocotyl), cultured them in vitro separately (Additional file 4: Figure S3), and measured their gossypol contents at 0, 2, 4, 8, 12 and 16 days after culture.

**Fig. 4:**
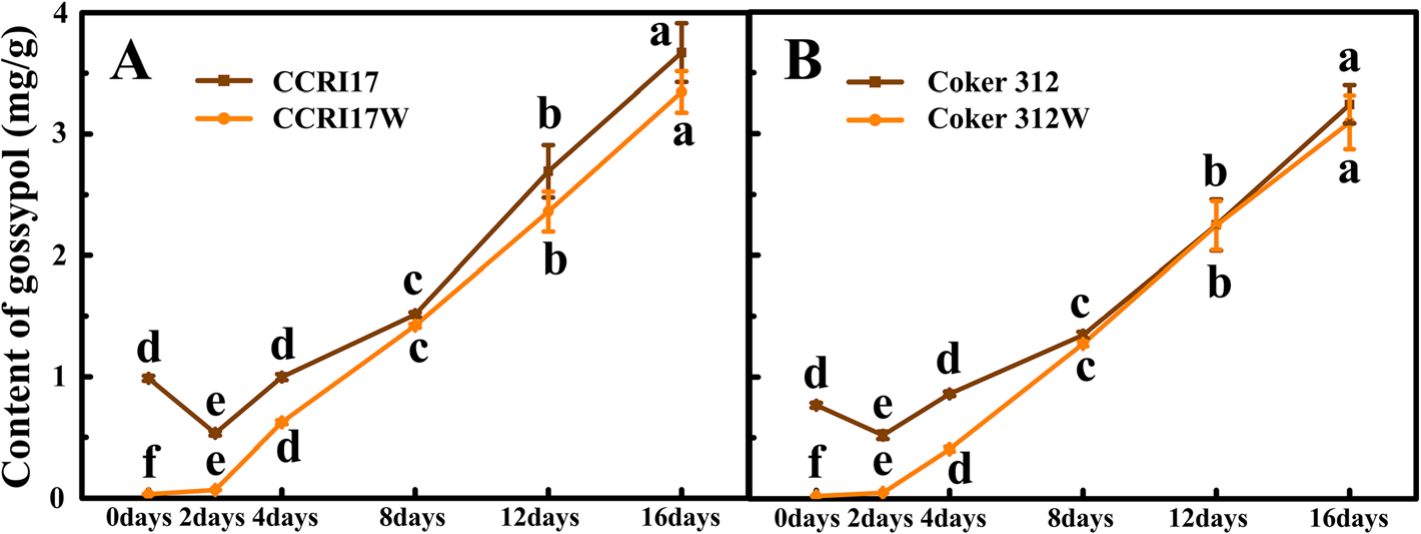
The dynamic changes of gossypol content in the root systems of two pairs of NILs during the root tip culture in vitro. Each sample was carried out in three biologicalrepetitions. Different letters indicate significant difference at the different times, *P* < 0.05

The initial gossypol content in the roots of the glanded cottons, CCRI17 and Coker 312, was 0.986 and 0.768 mg/g, respectively. At the beginning (2 days after incubation), their gossypol contents decreased by 46.06 and 32.22%, respectively, and then increased consistently. At 16 days of culture, they increased by 2.72 and 3.22 folds, respectively (Fig. 4a). However, the initial gossypol contents in the roots of two glandless cottons, CCRI17W and Coker 312 W were only 0.033 and 0.023 mg/g, respectively. A subsequent increase was observed for both genotypes throughout the culture period. At 16 days of culture, their gossypol contents increased by 133.47 and 100.42 folds, respectively, which were almost as high as those of their glanded NILs (Fig. 4b). These results strongly suggested the great ability of gossypol biosynthesis in both glanded and glandless cottons.

**Fig. 5:**
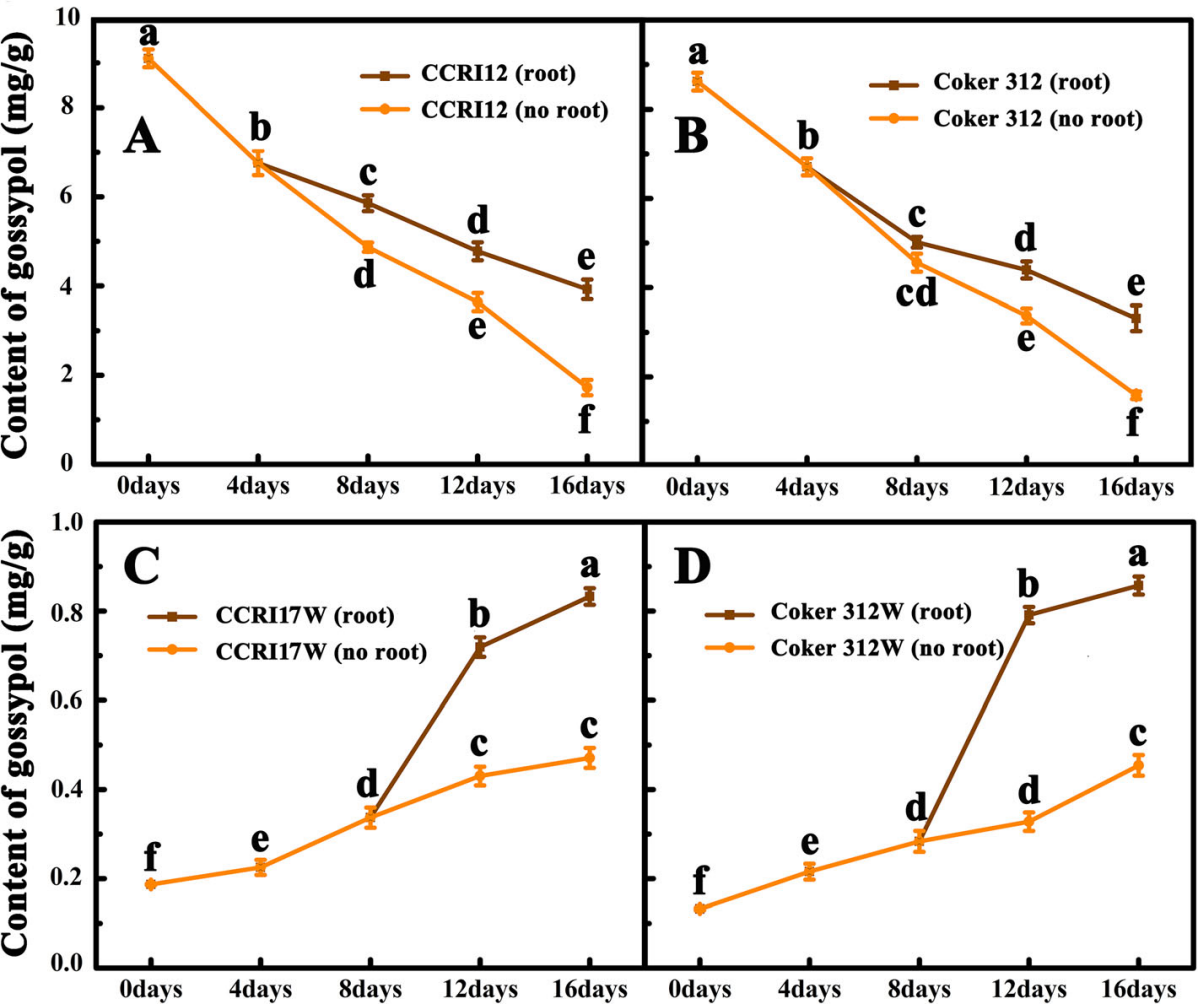
The dynamic changes of gossypol content in the rootless seedlings (with and without regenerated root) of two NILs. Each sample was carried out in three biological repetitions. Different letters in same genotype indicate significant difference at the different times, *P* < 0.05

Some in vitro cultured rootless seedlings produced regenerated roots at about four and 8 days after rootless seedling culture in the glanded and glandless NILs, respectively. The dynamic change of gossypol contents in the two types (with or without regenerated roots) of seedlings were investigated (Fig. 5). For the glanded cotton, gossypol contents in seedlings without regenerated roots showed a more significant decrease than those with regenerated roots (Fig. 5a and b). Compared with the gossypol contents of seedlings at the 4 days after culture, the gossypol contents of the seedlings with regenerated roots at 16 days after culture decreased to 58.06% (CCRI17) and 49.39% (Coker 312), while that of non-regenerated roots decreased to 25.51% (CCRI17) and 23.67% (Coker 312). For the glandless NILs, gossypol contents in the seedlings always increased throughout the culture period. The difference in gossypol content between the two types of seedlings could not be seen at the beginning of culture period (8 days) before root regeneration. But thereafter (8–16 days), a more profound increase of gossypol content was observed in the seedlings with regenerated roots as compared with the seedlings without regenerated roots. Finally, the gossypol content in the seedlings with regenerated roots was about twice the amount of that in non-regenerated roots (Fig. 5c and d). These results indicated that the cotton roots were the primary place of gossypol biosynthesis, but other organs might also have relatively weak ability to produce gossypol.

**Fig. 6:**
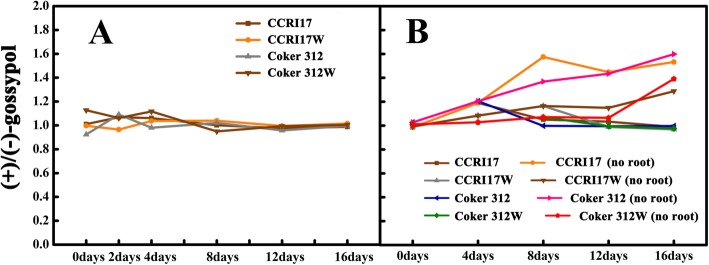
The ratio of (+)/(−)-gossypol in the growing period of organs culture. **a** Root culture. **b** Rootless seedling culture

We further measured the changes of gossypol isomers in the in vitro cultured roots and rootless seedlings (Additional file 5: Table S2 and Additional file 6: Table S3). The ratio of (+)-gossypol and (−)-gossypol in the in vitro cultured roots was approximately one during the whole culture period (Fig. 6a). However, for the in vitro cultured rootless seedlings, the ratio of (+)-gossypol and (−)-gossypol in the seedlings without regenerated roots was higher than that with regenerated roots (Fig. 6b). Therefore, it is implied that the gossypol produced by the root system is racemic gossypol, while the gossypol produced by other organs is optical gossypol.

### Expression patterns of the key genes in the gossypol biosynthetic pathway

**Fig. 7:**
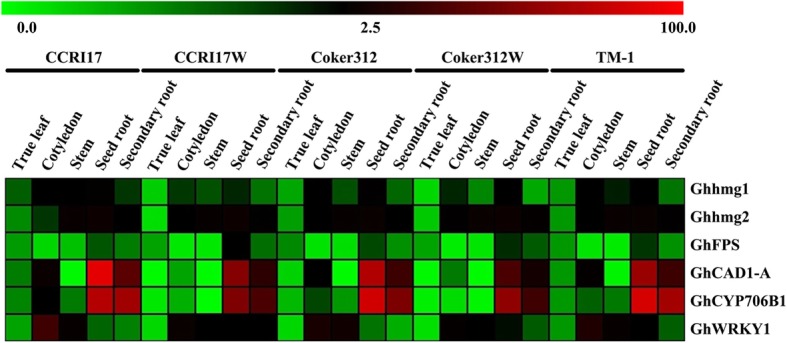
Expression patterns of the key genes participating in the pathway of gossypol biosynthesis in CCRI17, CCRI17W, Coker 312, Coker 312 W and TM-1. The color scales represent the relative signal intensity values

The expression levels of key genes involved in the gossypol biosynthesis pathway in CCRI17, CCRI17W, Coker 312, Coker 312 W and TM-1 were quantified (Fig. 7).

In upstream of the gossypol biosynthesis pathway, HMGR is considered as one of the most important enzymes [27]. The corresponding encoding genes, *hmg1* and *hmg2*, were highly expressed in the root systems of the five genotypes and the expression levels of *hmg2* in all tested tissues were higher in the glanded NILs than that of glandless plants. *FPS*, a gene for farnesyl pyrophosphate biosynthesis, had showed its highest expression level in the root system, followed by true leaves. The highest expression level of *CAD1-A* was also observed in the root system in all genotypes. *CAD1-A* showed higher expression level in seed root as compared with secondary roots. In the downstream of gossypol biosynthesis pathway, a key gene, *CYP706B1* [30]*,* also showed the highest expression level in the root system. Similar to *hmg2*, both *CAD1-A* and *CYP706B1* had a higher expression level in the glanded cottons than their glandless NILs in each tissue. In addition, *WRKY1*, a transcription factor related to gossypol biosynthesis [31], showed high expression levels in cotyledon and stem, and followed by roots. These results revealed that all the key genes, except *WRKY1*, in the gossypol biosynthesis pathway had a relatively high expression level in roots and a relatively low expression level in leaves. Therefore, it was suggested that seed root might be the major organ responsible for gossypol biosynthesis while leaves could play a very limited role in gossypol biosynthesis.

Our results showed that all the genes had determined a higher expression levels in the glanded NILs as compared with glandless NILs, while the dominant and recessive glandless accessions had a slightly different gene expression.

## Discussion

Gossypol is one of the most important secondary metabolites for cotton plant growth and development [39, 40]. However, the high content of gossypol in cottonseeds limited the broad utilization of cottonseeds that containing rich oil and protein. Thus, it is imperative to better understand gossypol metabolism. Through dynamic analysis of gossypol contents, plant grafting, organ culture and expression profile, the gossypol biosynthesis and accumulation were revealed in this research.

Specific secondary metabolites play a key role in their corresponding plant development [41]. It was reported that gossypol could promote the differentiation of somatic cells and that the glandless plant showed a better regeneration rate from somatic cells [42]. In our results of organ culture, the glanded rootless seedlings which had more gossypol generated adventitious roots significantly earlier than the rootless seedlings from the glandless NILs, which indicated a role of gossypol in root regeneration.

The mechanism of gossypol biosynthesis has been poorly understood. Previously, Smith showed gossypol biosynthesis in the excised root based on in vitro culture [38], but it was not showed whether or not other plant organs would also have the ability in biosynthesis of gossypol. In this study, through cotton grafting and organ culture, it was found that gossypol was mainly synthesized in the root system while other organs had a weaker ability in gossypol biosynthesis, and that both the glanded and glandless root systems had strong ability to synthesize gossypol although roots of the glanded cotton were more capable than those of the glandless cotton. Moreover, grafting between cotton and sunflower confirmed that the regenerated roots were able to synthesize gossypol that then transported to leaves. Higher expression levels of the key genes participating in the gossypol biosynthesis in roots than in other organs strongly supported that gossypol is mainly produced in the root system of both the glanded and glandless cotton plants. Besides, expression profile results also showed that the levels of all the key gene expressions in gossypol biosynthesis pathway were relatively low in leaves, indicating that leaves might have weak ability to synthesize gossypol. In addition, higher gene expression levels in seed roots than in secondary roots might suggest that seed roots having a stronger ability of gossypol biosynthesis than other kinds of roots.

The transportation of gossypol in cotton plant has remained unclear. Previous study showed that gossypol was transported and stored in the pigment glands [43]. Our results observed in the three tissues (cotyledon, root and leaf) indicated that gossypol contents in the roots of glanded NILs might be the result of in-situ biosynthesis and accumulation and /or transportation from cotyledons. While for the glandless NILs, gossypol might be synthesized only in roots and then transported to cotyledons, leaves and other organs. Specifically, at the seed germination stage, the primary source of gossypol was cotyledon. Roots started to synthesize and accumulate gossypol at the cotyledon flattening stage. At the later stages, gossypol was mainly synthesized and accumulated in roots and gradually transported into other organs. However, gossypol contents were quite different between the glanded and glandless plants due to the presence and absence of pigment glands. The level of gossypol content in the glanded plant was much higher than that in the glandless plant, because pigment glands in the former served as gossypol sinks. It seems that the absence of such sink may reduce the movement of gossypol from roots to other organs or maybe cause decomposition of gossypol after transportation. Based on our current results and previous published articles [38], it may suggest that gossypol in the glanded plants is mainly synthesized in roots and then transported to cotyledon and other organs, and eventually stored in the pigment glands. However, gossypol in glandless cotton is mainly synthesized and accumulated in roots, but transport only a limited part of gossypol to other organs or decompose due to lack of storage in pigment glands.

Our grafting experiments demonstrated that the dominant and recessive glandless NILs had little difference in their impact on gossypol content. It was suggested that the biosynthesis and accumulation of gossypol pathways were not related to the dominant and recessive glandless genes. Moreover, the grafting experiments with sunflower rootstocks further illustrated the importance of cotton roots in gossypol biosynthesis. However, according to the result that the gossypol had a key role in root regeneration in rootless seedling, whether the gossypol had a feedback effect on promoting root regeneration during grafting between cotton and sunflower remains unclear. Therefore, the exact role of gossypol in cotton growth and development need further investigation.

The relationship between gossypol and pigment glands is independent and related. According to gene silencing evidence, it was reported that gossypol biosynthesis and pigment gland formation could not directly be coupled [33]. They were controlled by different molecular mechanisms, but restraining formation of pigment glands had a countering effect on the biosynthesis and accumulation of gossypol. Besides, using RNAi of key genes to disrupt gossypol biosynthesis in cottonseed tissue, it was suggested that decrease of gossypol biosynthesis had an effect on the expression of pigment glands [2]. In addition, delayed gland morphogenesis trait has been reported in several wild cotton species from Australia*,* in which the pigment gland formed after seed germination, and then gossypol appeared [44, 45]. It was indicated that gossypol could only be accumulated after pigment glands formation. Our grafting experiments demonstrated that gossypol content could change without changing the expression of pigment glands, indicating that the pathways of gossypol biosynthesis and pigment gland formation were independent from each other. We also demonstrated that both glanded and glandless root system had the strong ability of gossypol biosynthesis, which indicating that gossypol biosynthesis was not tightly related to the expression of pigment glands. However, without pigment glands, gossypol could not be stored. Therefore, an appropriate explanation would be that gossypol is the material to be stored, and the pigment glands are the tissues of storage. Further investigations are necessary to elucidate the complicated relationship between gossypol and pigment glands.

Earlier studies have shown that (−)-gossypol had stronger biological activity than (+)-gossypol [22–24], and only (−)-gossypol was toxic to animals [11, 12]. Thus, by reducing the ratio of (+)-gossypol and (−)-gossypol without change of the total amount of gossypol, it is possible to increase the amount of cottonseed used as feed of ruminant or non-ruminants. According to our results of organ culture, we discovered that without the regenerated root, the (−)-gossypol content decreased faster than (+)-gossypol, while with the regenerated root, the (−)-gossypol content was almost the same with (+)-gossypol. Combine with the result of organ culture that root produced the same amount of (+)-gossypol and (−)-gossypol and rootless seedling produced more (+)-gossypol than (−)-gossypol, it was speculated that roots produce the racemic gossypol and other organs produce the optical gossypol. These findings may help clarify the major organ for biosynthesis of the optical active gossypol, but further studies are needed to investigate the specific roles of (+)-gossypol and (−)-gossypol in cotton plants and how their ratio can be changed genetically.

In current study, grafting and culturing of different organs were first used to study the metabolism of gossypol, which could be applicable in researching other metabolites. According to the previously reported compound synthesis [46, 47], technique of isotope labeling and tracking may be used to monitor the location of gossypol biosynthesis and transportation. Furthermore, as gossypol has the potential to be a medicine [19–21], organ culture in vitro may offer a novel method for the production of gossypol in pharmaceutical industry.

## Conclusions

Our study demonstrated that gossypol was primarily synthesized in the root systems of both the glanded and glandless cotton plants, while other organs also had a rather weaker ability of gossypol biosynthesis. Gossypol biosynthesis was not directly related to the expression of pigment glands, but the presence of pigment glands was essential for gossypol accumulation. The gossypol produced in cotton root was mainly racemic, while other organs synthesized the optically active gossypol. These findings not only shed light into the complex regulation network of gossypol metabolism, but would also accelerate the process towards breeding for cottons with enhanced commercial values.

## Methods

### Plant material and sampling

Two pairs of glanded and glandless upland cotton (*Gossypium hirsutum* L.) near isogenic lines, CCRI17 vs CCRI17W and Coker 312 vs Coker 312 W, and the genetic standard upland cotton line, TM-1, were used in the experiments. CCRI17 was developed by Institute of Cotton Research of CAAS, and Coker 312 and TM-1 were given by USDA-ARS, College Station, Texas, USA, and they were all deposited in National Cotton Germplasm Medium Bank. CCRI17W and Coker 312 W were developed by our lab and deposited in Cotton Germplasm Bank of Zhejiang University. CCRI17 and Coker 312 were glanded cottons, while CCRI17W and Coker 312 W were glandless cottons. The glandless phenotypes of CCRI17W and Coker 312 W were controlled by dominant gene (*Gle 2*) and recessive genes (*gl*_*2*_ and *gl*_*3*_), respectively. All the materials were kept by self-fertilization at Zhejiang University. Pigment glands were located in the root, shoot, leaves and seeds of glanded cotton. The sunflower (*Helianthus annuus* L.) cultivar Sandaomei (SDM) used as the rootstock material in this study was developed by Jilin Academy of Agricultural Sciences, China, and deposited in Germplasm Bank of Jilin Academy of Agricultural Sciences.

All the plant materials were grown in seedling pot (vermiculite: nutrition soil = 1: 1) at 28 ± 2 °C with natural light in the greenhouse of Zhejiang University. Water was applied once every 3 days.

### Plant grafting

Seedlings with two true leaves were used as the scions and rootstocks. The glanded cotton scions, TM-1, were grafted on glandless cotton rootstocks (CCRI17W and Coker 312 W) and sunflower rootstocks (SDM) by the method of improved bark grafting. Likewise, the glandless cotton scions, CCRI17W and Coker 312 W were also grafted on the glanded cotton rootstocks (TM-1).

Upon grafting in the watered soil, the grafted cotton seedlings were covered with the plastic bags and grown under 28 ± 2 °C. About 4 days later, four small holes were made on the plastic bags covering the seedlings for gas exchange. Two weeks later, the covering plastic bags were removed from the seedlings and the seedlings were grown as the normal plants afterward.

True leaves from the grafted cotton with sunflower rootstock were sampled from day 0 to day 12, and mature cottonseeds were sampled at the harvest stage (45 days post anthesis). All the samples were immediately frozen in liquid nitrogen and stored at − 80 °C for determination of gossypol contents.

### Organ culture in vitro

Cottonseeds were washed twice by sterile water for 3 min, then, disinfected twice by 70% alcohol (each for 3 min), followed by sterilization in mercuric chloride for 10 min, and finally washed five times in sterile water (each for 2 min). The kernels were germinated on the MS medium. When seed root attained to the length of 2 cm (Additional file 4: Figure S3A), the root (about 1 cm) was cut from the seedling for root culture in vitro (Additional file 4: Figure S3D) and the seedlings without root were cultured in vitro (Additional file 4: Figure S3B).

The medium used for root culture in vitro contains macro nutrients and 1/2 micro nutrients of the MS basic medium, vitamins and organic materials of B6 medium containing 100 mg/L of inositol, 1 mg/L of nicotinic acid, 10 mg/L of vitamin B1, 1 mg/L of vitamin B6, 20 mg/L of sucrose and 0.125 mg/L of IBA. Approximately 0.8% of agar was used as the solid material, and the pH of the medium was adjusted to 6.4. Growth conditions for root culture were 14/10 h day night intervals with light intensity of 2000 lx and average temperature of 28 ± 2 °C.

Rootless seedling culture in vitro was done similar to root culture, except slight change in the medium, in which the MS basic medium, vitamins and organic materials of B6 basic medium, 0.1 mg/L kinetin, and 20 mg/L of sucrose were used.

Cultured root and rootless seedling with and without regenerated root in vitro were sampled from day 0 to day 16. All the samples were immediately frozen in liquid nitrogen and stored at − 80 °C for determination of gossypol contents.

### Gossypol determination

Gossypol enantiomers were measured by High Performance Liquid Chromatography (HPLC). Standard gossypol solution was prepared by dissolving 0.01 g of HPLC-grade (±)-gossypol in 10 mL of acetonitrile. And then 0.001, 0.002, 0.005, 0.050, 0.100, 0.200, 0.500, 0.800, 1.000, 2.000 and 3.000 mL standard (±)-gossypol solutions were added into 2 mL derivative reagents containing 2% D-alaninol, 10% acetic acid and 88% acetonitrile, which function to created separable gossypol stereoisomers on reverse column. Then they were water bathed at 75 °C for 45 min and adjusted to 10 mL using acetonitrile for 0.05, 0.10, 0.25, 2.50, 5.00, 10.00, 25.00, 40.00, 50.00, 100.00 and 150.00 mg/L serial standard (+)-gossypol and (−)-gossypol preparations. These preparations were performed on HPLC to create the calibration curves for (+)-gossypol and (−)-gossypol measurement.

All samples were dried at 30 °C to constant weight, ground to powder with grinder, and stored at − 80 °C. An amount of 0.10 g from each sample was suspended into 2 mL derivative reagent to form sample solution. Sample solutions were water bathed at 75 °C for 45 min. Sample suspensions were filtered through quantitative filter paper followed by a filtration with a 0.45 μm syringe filter (Agela, Newark, USA). The sediment was washed three times by acetonitrile. After this procedure, the extract was adjusted to 10 mL using acetonitrile for sample preparations to be measured.

HPLC analysis were performed on Agilent 1100 (Agilent, Santa Clara, USA), equipped with an auto-sampler and an UV detection. A C18 column (250 mm × 4.6 mm, 5 μm, Dikma, Richmond Hill, USA) was employed as stationary phase. The mobile phase consisted of acetonitrile/0.2% H_3_PO_4_ (75/25, v/v). Injection volume was 10 μL and the flow rate was 1.0 mL/min. The UV detector was set at 238 nm and the temperature was 25 °C. Gossypol preparations were measured in three technical repetitions. The retention times of (+)-gossypol and (−)-gossypol were 10 min and 13.75 min, respectively.

### Image analysis

The pigment glands of the leaves were observed and taken as images through an Olympus dissecting microscope (LEICA MZ95, Germany) with a digital camera. Density and size of the pigment glands in the images were measured by the Image Pro Plus (V6.0) software.

### Quantitative RT-PCR

True leaves, cotyledons, seed roots, secondary roots and stems were sampled from cotton plants at the second true-leaf stage with three biological repetitions. All the samples were immediately frozen in liquid nitrogen and stored at − 80 °C. RNA of each sample was extracted using a Total RNA Extraction kit (Aidlab, Beijing, China). First strand cDNA was synthesized using TransScript One-Step gDNA Removal and cDNA Synthesis SuperMix (TransGen Biotec Co. Ltd.) following the manufactures protocol. Primers for qRT-PCR were designed with the Primer 5.0 software. All primers used in the experiment are listed in Additional file 7: Table S4 and cotton *UBQ7* was used as an internal control. The amplification reactions of qRT-PCR were performed with Lightcycler 96 system (Roche) using SYBR the premix Ex taq (TakaRa) with the following parameters: 30 s initializing denaturation at 95 °C; followed by 45 cycles of 10 s denaturation at 95 °C, 10 s annealing at 54 °C, and 20 s extension at 72 °C. In addition, the default setting for the melting curve stage was chosen. The relative expression levels were calculated by the method of 2^-ΔΔCt^. The heatmap for expression profiles was generated with the Mev 4.0 software.

### Statistical analysis

Statistical analysis for (±)-gossypol content and pigment glands was carried out by SPSS20.0. Data were represented as mean ± stand deviation (SD), and values of *P* < 0.05 and *P* < 0.01 were considered as statistically significant and extremely significant, respectively.

## Supplementary information

**Additional file 1: Figure S1.** The grafting combination of glanded and glandless cotton, sunflower and glanded cotton. (A) (B) showed the combination of glanded scion and glandless rootstock; (C) (D) showed the combination of glanded scion and sunflower rootstock.

**Additional file 2: Table S1**. The diameter and density of pigment glands in the leaves of the scions (TM-1) after grafting on different rootstocks^a^.

**Additional file 3: Figure S2.** Cotton scions with the generated roots 8 days after grafting on the rootstocks of sunflower.

**Additional file 4: Figure S3.** The processes of root culture and rootless seedling culture in vitro. (A) The germinated cottonseed was cut to a root and a rootless seedling; (B) The incubated rootless seedlings in the media; (C) The survived rootless seedlings; (D) The incubated root in the media; (E) The survived root systems.

**Additional file 5: Table S2.** The content (mg/g) of (±)-gossypol in the root systems at different times during the root culture in vitro^a^.

**Additional file 6: Table S3.** The content (mg/g) of (±)-gossypol in the plants at different times during the rootless plant culture in vitro^a^.

**Additional file 7: Table S4.** All primers used in this study.

## Data Availability

The datasets used and/or analysed during the current study are available from the corresponding author on reasonable request.
