# Supplementary material for: Cotton roots are the major source of gossypol biosynthesis and accumulation
Source: BMC Plant Biol. 2020 Feb 27;20:88. doi: 10.1186/s12870-020-2294-9 (PMC7045692; doi:10.1186/s12870-020-2294-9)
Supplement: Supplementary file 1 — Additional file 1: Figure S1. The grafting combination of glanded and glandless cotton, sunflower and glanded cotton. (A) (B) showed the combination of glanded scion and glandless rootstock; (C) (D) showed the combination of glanded scion and sunflower rootstock. [file 12870_2020_2294_MOESM1_ESM.pdf]

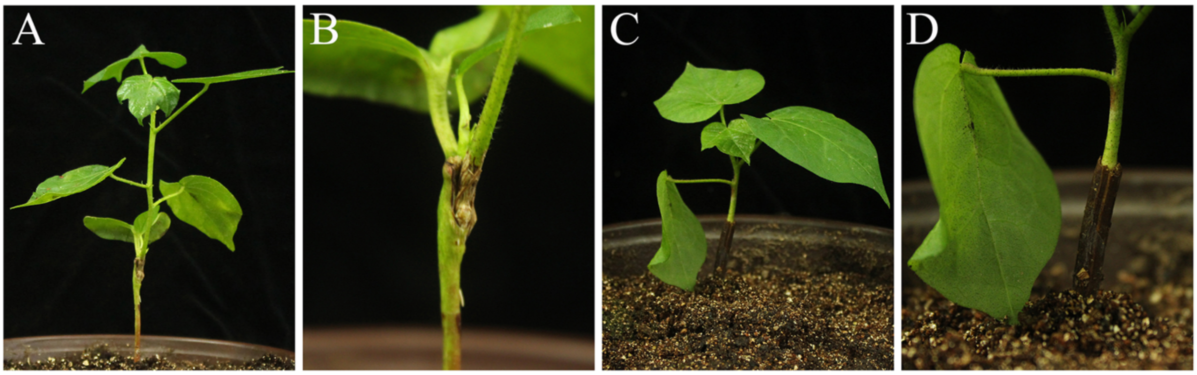

Figure S1. The grafting combination of glanded and glandless cottons, sunflower and glanded cotton. (A) and (B) show the combination of glanded scion and glandless rootstock; (C) and (D) showed the combination of glanded scion and sunflower rootstock.
