# Supplementary material for: Cotton roots are the major source of gossypol biosynthesis and accumulation
Source: BMC Plant Biol. 2020 Feb 27;20:88. doi: 10.1186/s12870-020-2294-9 (PMC7045692; doi:10.1186/s12870-020-2294-9)
Supplement: Supplementary file 2 — Additional file 2: Table S1. The diameter and density of pigment glands in the leaves of the scions (TM-1) after grafting on different rootstocksa. [file 12870_2020_2294_MOESM2_ESM.pdf]

Additional files

Cotton roots are the major source of gossypol biosynthesis and accumulation

Tianlun Zhao[1], Qianwen Xie[1], Cong Li[1], Cheng Li[1], Lei Mei[1], John Z. Yu[2], Jinhong Chen[1], Shuijin Zhu[1*],

* Correspondence: shjzhu@zju.edu.cn

[1]Department of Agronomy, Zhejiang University, Hangzhou 310058, Zhejiang province, China

[2]USDA-ARS, Southern Plains Agricultural Research Center, College Station, TX 77845, USA

Table S1. The diameter and density of pigment glands in the leaves of the scions (TM-1) after grafting on different rootstocks[a]

Traits[b]	Rootstocks				
	N[c]	TM-1	CRI17W	Coker 312W	SDM
**Dimeter (μm)**	119.01±12.13 Aa	119.32±13.32 Aa	117.69±12.40 Aa	118.36±12.65 Aa	117.51±10.86 Aa
**Density (Number/cm^2)**	97.45±4.40 Aa	98.53±5.4 Aa	96.57±5.72 Aa	96.75±5.29 Aa	97.27±5.70 Aa

[a]Values are mean ± standard deviation (SD). Letters behind the values in the same row indicate significant difference at different times. Lowercase and uppercase letters indicate significant at *P*=0.05 and 0.01 respectively.

[b]Diameter: the diameter of pigment glands; Density: the density of pigment glands.

[c]N: the normal TM-1 cotton plant without grafting.
