# Supplementary material for: Cotton roots are the major source of gossypol biosynthesis and accumulation
Source: BMC Plant Biol. 2020 Feb 27;20:88. doi: 10.1186/s12870-020-2294-9 (PMC7045692; doi:10.1186/s12870-020-2294-9)
Supplement: Supplementary file 3 — Additional file 3: Figure S2. Cotton scions with the generated roots 8 days after grafting on the rootstocks of sunflower. [file 12870_2020_2294_MOESM3_ESM.pdf]

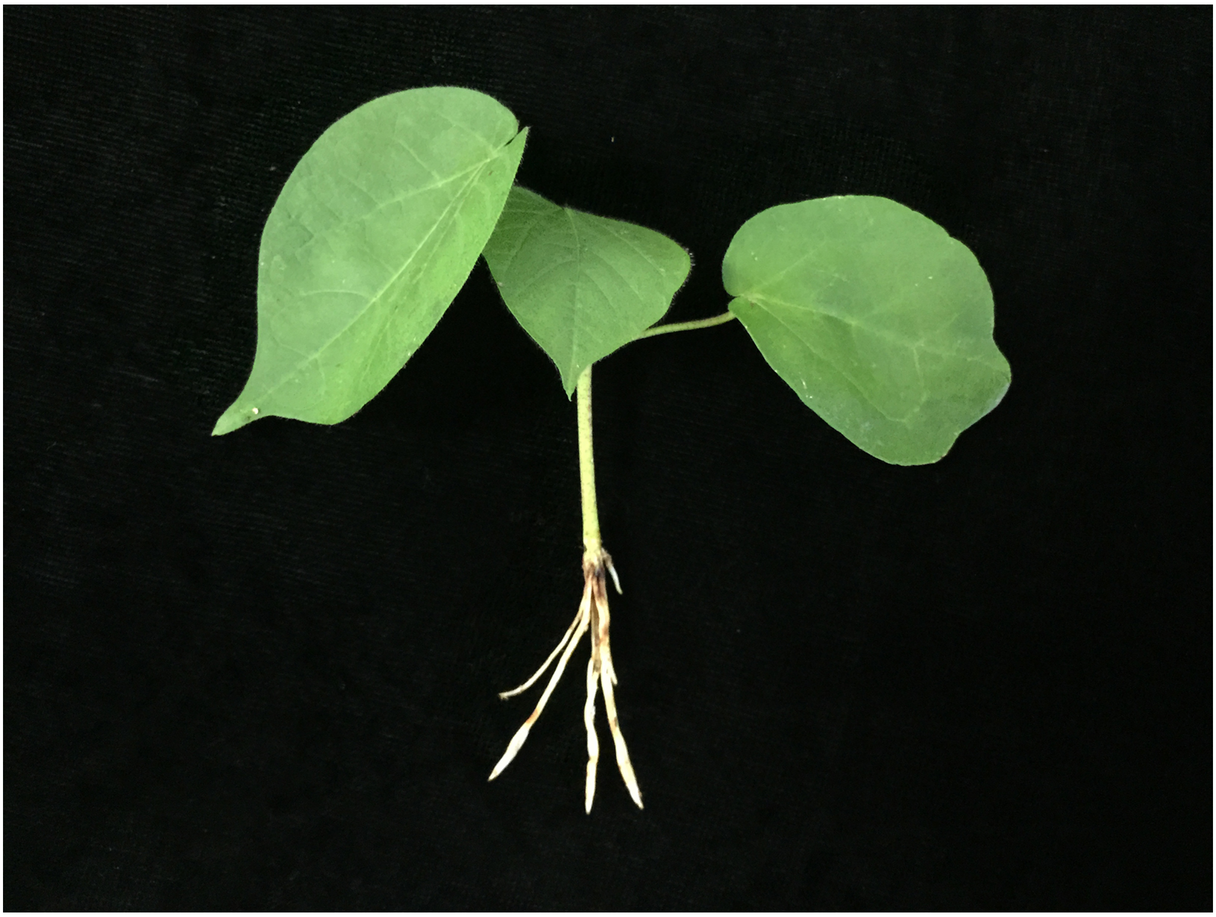

Figure S2. Cotton scions with the generated roots eight days after grafting on the rootstocks of sunflower.
