# Supplementary material for: Cotton roots are the major source of gossypol biosynthesis and accumulation
Source: BMC Plant Biol. 2020 Feb 27;20:88. doi: 10.1186/s12870-020-2294-9 (PMC7045692; doi:10.1186/s12870-020-2294-9)
Supplement: Supplementary file 4 — Additional file 4: Figure S3. The processes of root culture and rootless seedling culture in vitro. (A) The germinated cottonseed was cut to a root and a rootless seedling; (B) The incubated rootless seedlings in the media; (C) The survived rootless seedlings; (D) The incubated root in the media; (E) The survived root systems. [file 12870_2020_2294_MOESM4_ESM.pdf]

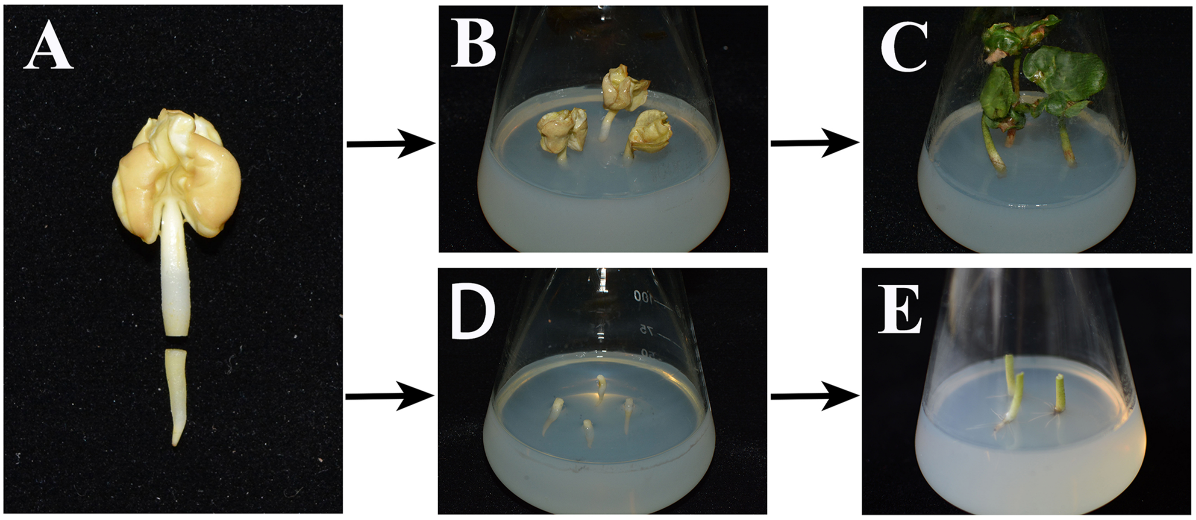

Figure S3. The processes of root tip culture and rootless seedling culture in vitro. (A) The germinated cottonseed was cut to a root and a rootless seedling; (B) The incubated rootless seedlings in the media; (C) The survived rootless seedlings; (D) The incubated root in the media; (E) The survived root systems.
