# Supplementary material for: Cotton roots are the major source of gossypol biosynthesis and accumulation
Source: BMC Plant Biol. 2020 Feb 27;20:88. doi: 10.1186/s12870-020-2294-9 (PMC7045692; doi:10.1186/s12870-020-2294-9)
Supplement: Supplementary file 5 — Additional file 5: Table S2. The content (mg/g) of (±)-gossypol in the root systems at different times during the root culture in vitroa. [file 12870_2020_2294_MOESM5_ESM.pdf]

Table S2. The content (mg/g) of (±)-gossypol in the root systems at different times during the root culture *in vitro*[a]

Cultivars	Traits	0 days[b]	2 days[b]	4 days[b]	8 days[b]	12 days[b]	16 days[b]
**CRI17**	**(+)-gossypol**	0.496±0.010 d	0.275±0.008 e	0.513±0.011 d	0.757±0.011 c	1.331±0.111 b	1.822±0.105 a
	**(-)-gossypol**	0.491±0.011 d	0.257±0.010 e	0.484±0.014 d	0.756±0.009 c	1.363±0.105 b	1.848±0.140 a
	**(±)-gossypol**	0.986±0.020 d	0.532±0.017 e	0.997±0.025 d	1.513±0.018 c	2.694±0.217 b	3.670±0.243 a
**CRI17W**	**(+)-gossypol**	0.017±0.001 f	0.033±0.001 e	0.318±0.010 d	0.724±0.010 c	1.178±0.100 b	1.686±0.100 a
	**(-)-gossypol**	0.017±0.001 f	0.034±0.001 e	0.306±0.007 d	0.697±0.007 c	1.183±0.066 b	1.660±0.070 a
	**(±)-gossypol**	0.033±0.002 f	0.068±0.002 e	0.624±0.017 d	1.421±0.016 c	2.361±0.165 b	3.347±0.170 a
**Coker312**	**(+)-gossypol**	0.369±0.009 d	0.271±0.018 e	0.426±0.009 d	0.680±0.010 c	1.101±0.115 b	1.615±0.083 a
	**(-)-gossypol**	0.399±0.011 d	0.249±0.014 e	0.435±0.010 d	0.666±0.018 c	1.149±0.095 b	1.627±0.122 a
	**(±)-gossypol**	0.768±0.019 d	0.520±0.031 e	0.861±0.018 d	1.346±0.027 c	2.250±0.210 b	3.241±0.156 a
**Coker312W**	**(+)-gossypol**	0.012±0.001 f	0.026±0.010 e	0.215±0.010 d	0.620±0.010 c	1.120±0.100 b	1.549±0.100 a
	**(-)-gossypol**	0.011±0.001 f	0.024±0.001 e	0.192±0.010 d	0.654±0.010 c	1.126±0.100 b	1.544±0.120 a
	**(±)-gossypol**	0.023±0.002 f	0.050±0.002 e	0.407±0.020 d	1.274±0.020 c	2.246±0.200 b	3.093±0.220 a

[a]Values are mean ± standard deviation (SD). Letters behind the values in the same row indicate significant difference at different times. Lowercase letter indicate significant difference, $p < 0.05$.

[b]days: days after the root incubation in the medium.
