# Supplementary material for: Cotton roots are the major source of gossypol biosynthesis and accumulation
Source: BMC Plant Biol. 2020 Feb 27;20:88. doi: 10.1186/s12870-020-2294-9 (PMC7045692; doi:10.1186/s12870-020-2294-9)
Supplement: Supplementary file 6 — Additional file 6: Table S3. The content (mg/g) of (±)-gossypol in the plants at different times during the rootless plant culture in vitroa. [file 12870_2020_2294_MOESM6_ESM.pdf]

Table S3. The content (mg/g) of (±)-gossypol in the seedlings at different times during the rootless seedling culture in *vitro* [a]

Cultivars	Traits	0days[b]	4days[b]	8days[b] (no root)	8days[b] (with root)	12days[b] (no root)	12days[b] (with root)	16days[b] (no root)	16days[b] (with root)
**CRI17**	**(+)-gossypol**	4.518±0.116 a	3.671±0.121 b	2.978±0.102 c	3.000±0.115 c	2.152±0.108 de	2.427±0.095 d	0.104±0.165 f	1.951±0.115 e
	**(-)-gossypol**	4.583±0.115 a	3.088±0.156 b	1.891±0.001 d	2.857±0.106 b	1.488±0.098 e	2.349±0.112 c	0.681±0.017 f	1.973±0.106 d
	**(±)-gossypol**	9.101±0.200 a	6.758±0.272 b	4.868±0.102 d	5.857±0.177 c	3.639±0.200 e	4.776±0.204 d	1.724±0.174 f	3.924±0.220 e
**CRI17W**	**(+)-gossypol**	0.093±0.001 f	0.117±0.009 f	0.181±0.011 e		0.230±0.010 d	0.358±0.011 b	0.265±0.011 c	0.410±0.009 a
	**(-)-gossypol**	0.094±0.001e	0.108±0.008 e	0.156±0.011 d		0.200±0.011 c	0.362±0.011 b	0.206±0.012 c	0.423±0.010 a
	**(±)-gossypol**	0.187±0.002 e	0.225±0.017 e	0.337±0.023 d		0.430±0.021 c	0.719±0.022 b	0.471±0.022 c	0.833±0.019 a
**Coker312**	**(+)-gossypol**	4.365±0.116 a	3.666±0.125 b	2.633±0.118 c	2.504±0.010 cd	1.985±0.108 e	2.190±0.082 d	0.977±0.072 f	1.655±0.176 e
	**(-)-gossypol**	4.250±0.085 a	3.044±0.078 b	1.925±0.089 e	2.512±0.112 c	1.384±0.067 ef	2.202±0.110 d	0.611±0.011 g	1.659±0.115 f
	**(±)-gossypol**	8.615±0.200 a	6.710±0.193 b	4.558±0.200 cd	5.016±0.121 c	3.369±0.168 e	4.392±0.191 d	1.588±0.083 f	3.314±0.288 e
**Coker312W**	**(+)-gossypol**	0.066±0.001 f	0.109±0.008 e	0.147±0.012 d		0.169±0.010 d	0.39457±0.010 b	0.264±0.012 c	0.423±0.011 a
	**(-)-gossypol**	0.066±0.001 f	0.106±0.011 e	0.137±0.012 d		0.159±0.011 d	0.396±0.009 b	0.190±0.012 c	0.434±0.010 a
	**(±)-gossypol**	0.132±0.002 f	0.216±0.018 e	0.284±0.023 d		0.329±0.021 d	0.791±0.018 b	0.454±0.023 c	0.858±0.020 a

[a]Values are mean ± standard deviation (SD). Letters behind the values in the same row indicate significant difference at different times. Lowercase letters indicate significant difference, $p < 0.05$.

[b]days: days after the rootless plant incubated in the medium.
