# Supplementary material for: Cotton roots are the major source of gossypol biosynthesis and accumulation
Source: BMC Plant Biol. 2020 Feb 27;20:88. doi: 10.1186/s12870-020-2294-9 (PMC7045692; doi:10.1186/s12870-020-2294-9)
Supplement: Supplementary file 7 — Additional file 7: Table S4. All primers used in this study. [file 12870_2020_2294_MOESM7_ESM.pdf]

Table S4. All primers used in this study

Purpose	Primer names	Sequence(5'-3')
qRT-PCR（*Ghhmg1*）	hmg1-F	CACCGGCGATCTTCGACTAA
qRT-PCR（*Ghhmg1*）	hmg1-R	AACCGCCGAGAAAAAGAGAGT
qRT-PCR（*Ghhmg2*）	hmg2-F	ACCCGTTATTGTTCTCAAGCCA
qRT-PCR（*Ghhmg2*）	hmg2-R	GAGCGTGAAGAACACCGCAT
qRT-PCR（*GhFPS*）	FPS-F	CGTCAATCGTCGCTCCGTTA
qRT-PCR（*GhFPS*）	FPS-R	AGCCACCATAAGCATCCTCG
qRT-PCR（*GhCAD1-A*）	CAD1-A-F	CGAAAATCGACCCAAGGCTG
qRT-PCR（*GhCAD1-A*）	CAD1-A-R	TGCAGCATCGATATCCGTATCA
qRT-PCR（*GhCYP706B1*）	CYP601B-F	ATTCCCGGTACATGCCGTTT
qRT-PCR（*GhCYP706B1*）	CYP601B-R	ATTTTCTTCACCGTCGGCCA
qRT-PCR（*GhWRKY1*）	WRKY1-F	CAACCCTTGTTTTCGCACCA
qRT-PCR（*GhWRKY1*）	WRKY1-R	GCGCTGACCCGATTCAATTC
